# Genetic variants in the *ZNF208* gene are associated with esophageal cancer in a Chinese Han population

**DOI:** 10.18632/oncotarget.13468

**Published:** 2016-11-19

**Authors:** Huijie Wang, Jianzhong Yu, Yanling Guo, Zhengxing Zhang, Guoqi Liu, Jingjie Li, Xiyang Zhang, Tianbo Jin, Zhaoxia Wang

**Affiliations:** ^1^ Department of Intergrated Traditional Chinese and Western Medicine in Oncology, Affiliated Luoyang Central Hospital, Zhengzhou University, Luoyang 471000, China; ^2^ Department of Neurology, Haikou People's Hospital, Haikou 570208, Hainan, China; ^3^ Key Laboratory of Resource Biology and Biotechnology in Western China, Ministry of Education, School of Life Sciences, Northwest University, Xi'an, Shaanxi 710069, China; ^4^ Xi'an Tiangen Precision Medical Institute, Xi'an, Shaanxi 710075, China

**Keywords:** ZNF208, esophageal cancer, single nucleotide polymorphisms (SNPs), association study

## Abstract

Previous studies showed an association between the *ZNF208* gene and gastric cancer. In this study, we investigated the association between single nucleotide polymorphisms (SNPs) in *ZNF208* and the risk of esophageal cancer in a Chinese Han population. We conducted a case-control study that included 386 cases and 495 controls. Five SNPs were selected from previous genome-wide association studies and genotyped using the Sequenom MassARRAY platform. Unconditional logistic regression was used to calculate odds ratios and 95% confidence intervals after adjustment for age and gender. Logistic regressionl analysis showed that two SNPs (rs8103163 and rs7248488) were associated with an increased risk of esophageal cancer under different inheritance models after Bonferroni correction. Haplotype analysis suggested that the four variants comprised one block, and that the G_rs2188972_C_rs2188971_C_rs8103163_C_rs7248488_ haplotype was significantly correlated with an increased risk of esophageal cancer. Our data indicate that variants in *ZNF208* are contribute to the susceptibility to esophageal cancer in a Chinese Han population.

## INTRODUCTION

Esophageal carcinoma is the sixth leading cause of cancer-related mortality worldwide [1]. Previous studies have demonstrated that China has the highest incidence of this aggressive malignancy [2]. The incidence rate was 186.34/10^5^ person-years in China [3] compared to 8.34/10^5^ person-years among Caucasian men in the U.S. [1]. The main risk factors for esophageal cancer are gastroesophageal reflux disease, smoking, and obesity [4–7]. However, only a subset of individuals who are exposed to these environmental risk factors actually develop esophageal cancer, suggesting that others factors (e.g. heritable) may also impact susceptibility.

Single nucleotide polymorphisms (SNPs) that are correlated with an increased risk of esophageal cancer have been identified previously [8–10]. The *Zinc finger protein 208 (ZNF208)* gene is located in the p12 region of human chromosome 19. ZNF208 is a member of the Zinc finger family of proteins, which bind to DNA through a series of zinc finger motifs and regulate gene transcription [11, 12]. Mutations in *ZNF208* have been observed in gastric cancer. Thus, it may act as a tumor suppressor [13]. This gene was also associated with the response to imatinib mesylate treatment in patients with gastrointestinal stromal tumor [14]. Recently, an association between one variant (rs8105767) within the *ZNF208* gene and telomere length was identified in a genome-wide association study. Interestingly, r s8105767 was associated with the risk of neuroblastoma, but not osteosarcoma or leukemia [15]. This SNP was also associated with the risk of lung adenocarcinoma, but not colorectal, breast, or prostate cancer [16]. It was also not associated with chronic lymphocytic leukemia or glioma [17, 18]. This variant was associated with reduced telomere length and coronary artery disease in a European population [19]. However, this SNP was not associated with reduced telomere length in squamous cell carcinoma of the head and neck in a Chinese Han population [20], and it was also not associated with gastric cancer susceptibility [21].

We performed a case-control study to evaluate the association between SNPs/haplotypes in the *ZNF208* gene and esophageal cancer susceptibility in a Chinese Han population. Fivevariants (rs2188972, rs2188971, rs8103163, rs7248488 and rs8105767) were included in the analysis. Our data. provide evidence for a correlation between SNPs in the *ZNF208* gene and esophageal cancer in the Chinese Han population.

## RESULTS

### Characteristics of the study population

We enrolled 385 esophageal cancer patients and 495 healthy controls in the study. The mean age was 60.68 ± 8.95 years in the case group and 54.48 ± 9.44 years in the control group. There were 300 men (79.8%) and 78 women (20.2%) in the case group, and 180 men (36.4%) and 315 women (63.6%) in the control group. There were no significant differences in age and gender between the cases and the controls (*p* < 0.001).

### Associations between the five SNPs in ZNF208 and the risk of esophageal cancer

The five SNPs that were genotyped are shown in Table 1. All SNPs were in Hardy-Weinberg equilibrium (HWE) in the control samples. The SNP distribution frequencies were calculated according to the genotyping data for the case and control groups. The minor allele frequencies are also shown in Table 1. All the alleles of the SNPs had non-significant Chi-square (χ2) values.

**Table 1:** Allele frequencies in cases and controls and odds ratio estimates for esophageal cancer

SNP_ID	Position	Location	Allele A^a^/B	MAF		HWE*p*-value	ORs	95%CI	*p*-value
				Case	Control				
rs2188972	22149458	3′utr	A/G	0.52	0.49	1	1.1	0.91-1.32	0.342
rs2188971	22152182	3′utr	T/C	0.32	0.3	0.593	1.08	0.88-1.32	0.488
rs8103163	22174752	intron	A/C	0.33	0.3	0.671	1.13	0.92-1.38	0.254
rs7248488	22188709	intron	A/C	0.33	0.3	0.595	1.13	0.92-1.38	0.261
rs8105767	22215441	intron	G/A	0.29	0.299	0.237	0.95	0.77-1.17	0.644

MAF, minor allele frequency; HWE: Hardy-Weinberg Equilibrium; ORs, odds ratios; CI, confidence interval.

^a^, Minor allele.

We identified associations between four SNPs in *ZNF208* and esophageal cancer using a logistic regression model after adjustment for age and gender. The detailed genotype distributions under various models are presented in Table 2. Interestingly, rs2188972 was associated with an increased risk of esophageal cancer in homozygote (*p* = 0.046) and additive (*p* = 0.046) models. Additionally, rs2188971 demonstrated a significant association in dominant (*p* = 0.037) and additive (*p* = 0.034) models. Rs8103163 showed a significant association in dominant (*p* = 0.009), additive (*p* = 0.008), and co-dominant models (homozygote *p* = 0.037, heterozygote *p* = 0.024). A significant association was also detected between rs7248488 and the risk of esophageal cancer in dominant (*p* = 0.017) and additive (*p* = 0.01) models, as well as in the co-dominant model for the homozygous genotype (*p* = 0.028). We did not observe any association between rs8105767 and esophageal cancer risk in any of the models. Only two mutations, rs8103163 (dominant *p* = 0.045; additive *p* = 0.040) and rs7248488 (additive *p* = 0.050), were associated with an increased risk of esophageal cancer after Bonferroni correction.

**Table 2:** Logistic regression analysis of the association between SNPs and esophageal cancer risk

SNP	Model	Genotype	Case	Control	OR(95%CI)	P* value
**rs2188972**	Co-dominat	GG	92	129		
	Heterozygote	AG	192	248	1.25(0.87-1.81)	0.232
	Homozygote	AA	101	118	1.55(1.01-2.37)	**0.046**
	Dominant	GG	92	129		
		AG-AA	293	366	1.34(0.95-1.90)	0.097
	Recessive	AG-GG	284	377		
		AA	101	118	1.33(0.94-1.89)	0.108
	Log-additive				1.24(1.00-1.54	**0.046**
**rs2188971**	Co-dominat	CC	179	243		
	Heterozygote	TC	163	202	1.35(0.98-1.86)	0.069
	Homozygote	TT	39	47	1.57(0.92-2.68)	0.101
	Dominant	CC	179	243		
		TC-TT	202	249	1.39(1.02-1.88)	**0.037**
	Recessive	TC-CC	342	445		
		TT	39	47	1.36(0.81-2.27)	0.24
	Log-additive				1.29(1.02-1.63)	**0.034**
**rs8103163**	Co-dominat	CC	170	243		
	Heterozygote	AC	167	205	1.45(1.05-2.01)	**0.024**
	Homozygote	AA	40	47	1.77(1.03-3.03)	**0.037**
	Dominant	CC	170	243		
		AC+AA	207	252	1.51(1.11-2.05)	**0.009**
	Recessive	AC+CC	337	448		
		AA	40	47	1.48(0.89-2.46)	0.135
	Log-additive				1.37(1.09-1.74)	**0.008**
**rs7248488**	Co-dominat	CC	175	243		
	Heterozygote	AC	166	204	1.38(0.99-1.90)	0.0503
	Homozygote	AA	43	48	1.8(1.06-3.04)	**0.028**
	Dominant	CC	175	243		
		AC+AA	209	252	1.45(1.07-1.97)	**0.017**
	Recessive	AC+CC	341	447		
		AA	43	48	1.54(0.93-2.54)	0.09
	Log-additive				1.36(1.08-1.71)	**0.01**
**rs8105767**	Co-dominat	AA	195	236		
	Heterozygote	GA	156	219	0.89(0.65-1.21)	0.45
	Homozygote	GG	33	38	1.44(0.81-2.58)	0.218
	Dominant	AA	195	236		
		GA+GG	189	257	0.95(0.71-1.29)	0.76
	Recessive	GA+AA	351	455		
		GG	33	38	1.53(0.87-2.68)	0.142
	Log-additive				1.05(0.82-1.33)	0.705

* Adjusted for age and gender.

### Association between haplotypes in ZNF208 and esophageal cancer

**Figure 1:**
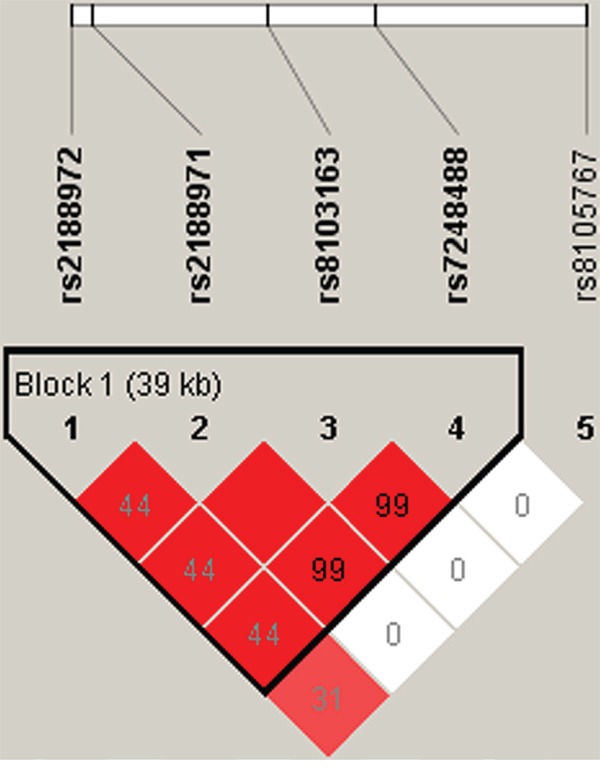
Linkage disequilibrium (LD) plots containing five SNPs from ZNF208

We performed linkage disequilibrium analysis for the five SNPs in the logistic regression model. One block was detected based on the calculated D' values (Figure 1). Three common haplotypes are shown in Table 3. We found that the A_rs2188972_ T_rs2188971_ A_rs8103163_ A_rs7248488_ haplotype was associated with a significantly increased risk of esophageal cancer (odds ratio [OR] = 1.33, 95% confidence interval [CI] 1.05–2.38, *p* = 0.031). An additional haplotype, G_rs2188972_ C_rs2188971_ C_rs8103163_ C_rs7248488_, which consisted of the major allele of all four SNPs, reached statistical significance (*p* = 0.031, OR = 0.79, 95% CI 0.64–0.98). Interestingly, this haplotype had a protective effect against esophageal cancer.

**Table 3:** The haplotype frequencies of *ZNF208* polymorphisms and esophageal cancer risk

rs2188972	rs2188971	rs8103163	rs7248488	Case%	Control%	OR(95%CI)	p* value
G	C	C	C	48.56	50.91	0.79(0.64-0.98)	**0.031**
A	C	C	C	18.64	18.79	0.94(0.71-1.25)	0.69
A	T	A	A	32.15	30.1	1.33(1.05-2.38)	**0.018**

* Adjusted for age and gender.

## DISCUSSION

We assessed the relationship between SNPs in the *ZNF208* gene and esophageal cancer in a Chinese Han population. Our results indicate that four SNPs (rs2188972, rs2188971, rs8103163, and rs7248488) are associated with an increased risk of esophageal cancer after adjustment for age and gender. However, we did not detect an association between rs8105767 and esophageal cancer susceptibility. This SNP was previously shown to be associated with reduced telomere length in a European population [19]. Several studies have provided evidence that telomere shortening increases the risk of esophageal carcinoma [10, 22–24]. However, rs8105767 showed no significant association with telomere length or gastric cancer in a Chinese population [21, 25], and it was not associated with esophageal cancer in our study. Thus, this SNP may not affect telomere length. Alternatively, the results may be attributed to differences in genetic background between the Chinese Han and European populations.

Zinc finger proteins are the largest family of transcriptional regulators. These proteins have critical roles in cell differentiation and development. There is evidence that Zinc finger proteins are broadly involved in tumorigenesis [26, 27]. Wang et al. [26] showed that ZNF300 promoted carcinogenesis by modulating the NF-γB pathway. Additionally, overexpression of ZNF300 stimulated cancer cell proliferation *in vitro* and significantly enhanced tumor development and metastasis in a xenograft mouse model. Another study demonstrated that ZNF280B promotes prostate cancer cell growth and survival through down-regulation of *p53* [28]. Wang et al. determined that the ZNF motif (Cys2-His2) in ZNF425 was the main region responsible for the transcriptional repression activity of this protein [29].

The conserved C2H2 motif is also present in ZNF208, suggesting that it may be involved in cancer development and progression. Rink et al. [14] found that the expression of the *ZNF43*, *ZNF208*, and *ZNF91* genes was correlated with the response to imatinib mesylate in patients with gastrointestinal stromal tumor. Depletion of any of the genes could alter treatment response. In another study of gastric cancer [13], the activity and mRNA expression of *ZNF208* were reduced as a consequence of somatic mutations, suggesting ZNF208 has a central role in tumor suppression. In our study, rs2188972, rs2188971, rs8103163, and rs7248488 were associated with the risk of esophageal cancer. All four SNPs are either intronic or 3′ UTR variants, which do not alter the coding sequence of the protein (Table 2). However, these SNPs may regulate transcription and affect gene expression.

Given the limited value of analysis of a single SNP, we evaluated the association between haplotypes in the *ZNF208* gene and esophageal cancer susceptibility. The ATAA haplotype accounted for 32.15% (case group) and 30.1% (control group) of all possible marker combinations (Table 3). After adjustment for gender and age, the results showed that the ATAA haplotype was associated with a significantly increased risk of esophageal cancer.

Although our study has provided evidence for an association between genetic variants in *ZNF208* and esophageal cancer, the results should be interpreted with caution. A post-hoc power analysis showed that all of the SNPs failed to reach the minimum level of statistical power (75%) for a case-control study. Thus, although the differences difference were statistically significant, the results must be verified in studies with larger sample sizessize. Our study had several limitations. First, selection bias and the limited sample size may have increased the number of false positive associations. Second, many other risk factors for esophageal cancer (e.g., smoking, alcohol consumption, pathology) were not included in the analysis. Finally, we did not analyze the biological functions of the mutations, which should be investigated in future studies.

In conclusion, our data indicate that rs2188972, rs2188971, rs8103163, and rs7248488 in the *ZNF208* gene as well as the ATAA haplotype are associated with an increased risk of esophageal cancer in a Chinese Han population.

## MATERIALS AND METHODS

### Study population and sample collection

Patient data and venous blood samples were collected starting in May 2014 at the First Affiliated Hospital of Xi'an Jiaotong University. A total of 386 cases and 495 controls were enrolled in our study. The case group consisted of inpatients who were diagnosed with pathologically verified esophageal cancer. All subjects participated in the screening program by undergoing an endoscopic staining examination with 1.2% iodine solution, and biopsies were collected from unstained areas of the mucosa. Tissue sections were reviewed by two experienced pathologists in order to ensure tumor cell purity > 50% and confirm the histological type. None of the patients had a history of other cancers or had received chemotherapy or radiotherapy. Patients who had comorbidities such as diabetes mellitus, hypertension, or any endocrine disorders were excluded. The controls were cancer-free individuals recruited from the health checkup center at the same hospital. Patient demographic data were also collected. All subjects were genetically unrelated Han Chinese and local residents of Northwest China.

The study was approved by the Clinical Research Ethics of Northwest University and Xi'an Jiaotong University. Written informed consent was obtained from all participants. Venous blood samples (5 mL) were collected from each subject into tubes containing EDTA, centrifuged, and stored at -80°C.

### Genotyping

Few studies have investigatedinvestigate the associations between SNPs in the *ZNF208* and esophageal cancer. We searched Pubmed and the Hapmap database for *ZNF208* SNPs with a MAF > 0.05 in the Chinese Han population. Five SNPs (rs2188972, rs2188971, rs8103163, rs7248488, and rs8105767) were selected for our study. Genomic DNA was extracted from the whole blood samples using the GoldMag-Mini Purification Kit (GoldMag Co. Ltd. Xian, China) according to the manufacturer's instructions. DNA concentrations were measured using a NanoDrop 2000 (Thermo Scientific, Waltham, Massachusetts, USA). SNPs were genotyped using the Sequenom MassARRAY RS1000 and the manufacturer's protocol [30]. The Sequenom MassARRAY Assay Design 3.0 software was used to design a multiplexed SNP MassEXTEND assay [31]. The Sequenom Typer 3.0 Software (Sequenom, Inc) was used for data management and analyses [30, 31].

### Statistical analysis

Genotype frequencies in the control subjects were tested for departure from HWE using χ2 tests. The genotype and allele distributions in the esophageal cancer patients and control subjects were compared using χ2 tests. ORs and 95% CIs were calculated by unconditional logistic regression analysis in co-dominant, additive, recessive, and dominant models after adjustment for confounding factors such as gender and age [32]. A two-sided *p* < 0.05 was considered statistically significant. The statistical analyses above were performed using Microsoft Excel and SPSS 20.0 (SPSS Inc., Chicago, IL, USA). Linkage disequilibrium and haplotypes were analyzed with the SHEsis software [33]. The Sampsize platform (http://sampsize.sourceforge.net/iface/s3.html) was used to evaluate the statistical power of this case-control study.
